# Mr. James Braid

**Published:** 1860-07

**Authors:** 


### NECROLOGICAL NOTICES.

MR. JAMES BRAID, of Manchester, whose name has of late been so much connected with the subject of hypnotic anæsthesia, died on the twenty-fifth of March, in the sixty-fifth year of his age, of an affection of the heart. In 1842 he investigated very fully and fairly the phenomena produced by animal magnetism and mesmerism, and proved that there was no such thing as animal magnetism, but grouped these phenomena under the terms, hypnotism, neuro-hypnotism, or nervous sleep. He was the author of several works on "Nervous Sleep," "Trance," and "Electro-Biology," which appeared between the years 1843 and 1852; and in 1855 he issued his "Observations on the Nature and Treatment of certain Forms of Paralysis." He also contributed many articles to medical periodicals, and some of his treatises have been translated into the German and French languages.

